# ﻿Three new species of the genus *Cicurina* Menge, 1871 from Chongqing, China (Araneae, Cicurinidae)

**DOI:** 10.3897/zookeys.1248.151580

**Published:** 2025-08-08

**Authors:** Hui-Yi Chen, Lu-Yu Wang, Zhi-Sheng Zhang, Feng Zhang

**Affiliations:** 1 Key Laboratory of Zoological Systematics and Application, College of Life Sciences, Hebei University, Baoding 071002, China; 2 Hebei Basic Science Center for Biotic Interaction, Hebei University, Baoding 071002, China; 3 Key Laboratory of Eco–environments in Three Gorges Reservoir Region (Ministry of Education), School of Life Sciences, Southwest University, Chongqing 400715, China

**Keywords:** Araneomorph spiders, biodiversity, description, morphology, southwestern China, taxonomy

## Abstract

Three new species of the spider genus *Cicurina* Menge, 1871, *C.jinyun***sp. nov.** (♂♀), *C.yinhe***sp. nov.** (♂♀) and *C.zhangfui***sp. nov**. (♂), are described from Chongqing, China. Morphological descriptions, photographs, and illustrations of copulatory organs are provided. The number of documented *Cicurina* species in Chongqing increases from 10 to 13.

## ﻿Introduction

The spider genus *Cicurina* Menge, 1871 has a complex taxonomic history, having been moved from Agelenidae to Dictynidae (Lehtinen, 1967), then to Hahniidae (Wheeler et al. 2017), before finally being placed in its own designated family, Cicurinidae (Gorneau et al. 2023). It comprises 86% (154 out of 179) of the species in the entire family, predominantly distributed across the Holarctic region, of which 114 species are distributed in North America (WSC 2025).

*Cicurina* spiders, characterized by their small size and secretive behavior, are typically found in leaf litter, under stones, and in caves, where they construct intricate small funnel webs that function as both shelters and hunting grounds (Gertsch 1992; Wang et al. 2019; Liao et al. 2022). Recent studies suggest that its diversity has been significantly underestimated, particularly in China where 31 species (10 in Chongqing Mun., 8 in Guizhou Prov., 5 in Hunan Prov., 3 in Guangdong Prov., 2 in Anhui Prov., 2 in Hubei Prov., 1 in Zhejiang Prov.) have been recorded (Chen 1986; Wang and Xu 1989; Song and Kim 1991; Wang 1994; Peng et al. 1996; Yin et al. 2012; Li and Wang 2017; Wang et al. 2019; Liao et al. 2022; Wang et al. 2024; Wang and Zhang 2025; WSC 2025). Based on our field experience, we speculate that numerous unknown *Cicurina* species likely exist in southern China, particularly in southwestern mountain areas.

In this paper, we describe and illustrate three new *Cicurina* species from Chongqing Municipality, China, with living specimen photos of two species. This work represents the third contribution to the study of *Cicurina* spiders in Chongqing, following Wang et al. (2024) and Wang and Zhang (2025).

## ﻿Material and methods

All specimens were preserved in 75% ethanol and examined, illustrated, photographed and measured using a Leica M205A stereomicroscope equipped with a drawing tube, a Leica DFC450 camera and LAS software (ver. 4.6). Male palps and epigynes were examined and illustrated after they were dissected. Epigynes were cleared by immersing them in pancreatin (Álvarez-Padilla and Hormiga 2007). Eye sizes were measured as the maximum dorsal diameter. Leg measurements are shown as: total length (femur, patella and tibia, metatarsus, tarsus). All measurements are in millimetres. Specimens examined here are deposited in the spider collection at the School of Life Sciences, Southwest University, Chongqing, China (**SWUC**).

Abbreviations used in the text: ALE, anterior lateral eye; AME, anterior median eye; MOA, median ocular area; PLE, posterior lateral eye; PME, posterior median eye.

## ﻿Taxonomy

### ﻿Family Cicurinidae Kishida, 1955 (洞叶蛛科)

**Genus *Cicurina* Menge, 1871**

**Figure 1.**
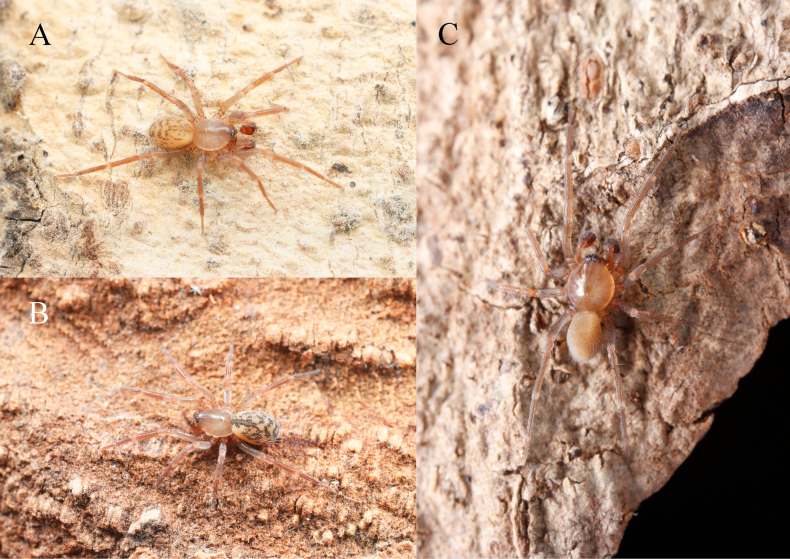
Photos of living specimens. A. *Cicurinajinyun* sp. nov., paratype male; B. *Cicurinajinyun* sp. nov., paratype female; C. *Cicurinayinhe* sp. nov., paratype male. Photographed by Qian-Le Lu.

**Figure 2.**
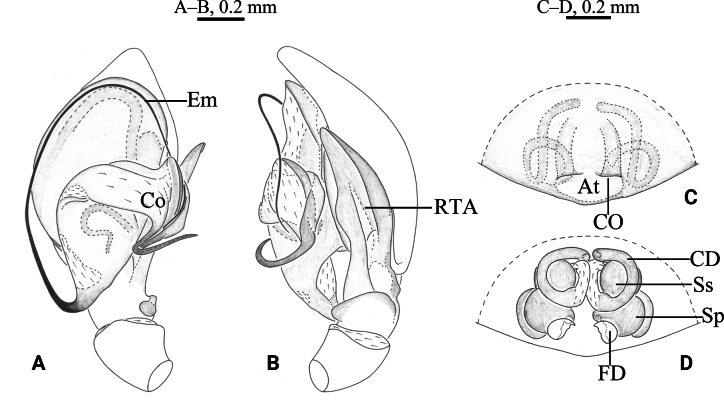
*Cicurinajinyun* sp. nov. holotype male (A, B) and paratype female (C, D). A. Left male palp, ventral view; B. Same, retrolateral view; C. Epigyne, ventral view; D. Vulva, dorsal view. Abbreviation: At = atrium; CD = copulatory duct; CO = copulatory opening; Co = conductor; Em = embolus; FD = fertilization duct; RTA = retrolateral tibial apophysis; Sp = spermathecae; Ss = secondary spermathecae.

**Figure 3:**
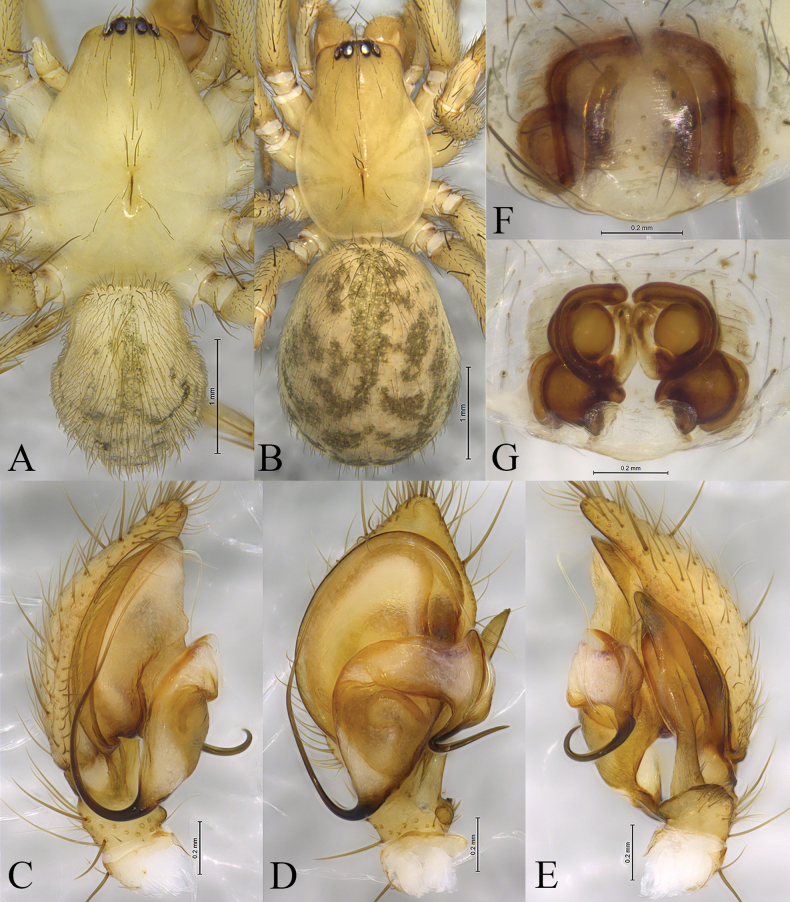
*Cicurinajinyun* sp. nov. holotype male (A, C–E) and paratype female (B, F, G). A. Male habitus, dorsal view; B. Female habitus, dorsal view; C. Left male palp, prolateral view; D. Same, ventral view; E. Same, retrolateral view; F. Epigyne, ventral view; G. Vulva, dorsal view.

Figs 1A–B, 2A–D, 3A–E, 8

洞叶蛛属

#### ﻿ Cicurina jinyun sp. nov.

Taxon classificationAnimaliaAraneaeCicurinidae

31418523-1FF8-59A8-9D34-023320E55732

https://zoobank.org/62DA73D2-1E3B-48ED-B31D-62822F2F265E

##### Type material.

***Holotype*** • male (SWUC-T-CI-13-01), China, Chongqing, Beibei, Jinyun Mountain National Nature Reserve, Baiyun Temple, 29°49'39"N, 106°22'59"E, elev. 641 m, 18 September 2016, L.Y. Wang leg. ***Paratypes***: • 6 males and 7 females (SWUC-T-CI-13-02–14), Jinyun Mountain National Nature Reserve, Beiwenquan, 29°51'32"N, 106°24'41"E, elev. 229 m, 30 December 2024, L.Y. Wang, M. Irfan, Y.N. Mu, X.Y. Zhang and E.X. Liu leg.

##### Etymology.

The specific name refers to the type locality.

##### Diagnosis.

The male of this new species is similar to that of *C.yuelushanensis* Wang, Zhou & Peng, 2019 (Wang et al. 2019: figs 7A, 8C–E) in having the strong retrolateral tibial apophysis, and the slender and filiform embolus, but differs by the longer and curved conductor end (Figs 2A, B, 3C–E) (vs. knife-like in *C.yuelushanensis*). The female is similar to that of *C.tetragongylodes* Liao, Yin, He & Xu, 2022 (Liao et al. 2022: 485, figs 6A–E, 7A, B) in having the posteriorly located epigynal atrium, the ball-shaped spermathecae and secondary spermathecae, but differs by the unconnected anterior edge of atrium (Figs 2C, 3F) (vs. closely connected in *C.tetragongylodes*) and the secondary spermathecae encircled by copulatory ducts (Figs 2D, 3G) (vs. all spermathecae surrounded by copulatory ducts in *C.tetragongylodes*).

##### Description.

**Male holotype** (Fig. 3A) total length 4.03. Prosoma 2.36 long, 1.72 wide; opisthosoma 1.73 long, 1.38 wide. Carapace pyriform, pale yellow, with several rows of setae. Eye sizes and interdistances: AME 0.03, ALE 0.11, PME 0.09, PLE, 0.12; AME–AME 0.04, AME–ALE 0.03, PME–PME 0.10, PME–PLE 0.05, ALE–PLE 0.04. MOA 0.24 long, anterior width 0.09, posterior width 0.27. Clypeus height 0.20. Chelicerae with three promarginal and nine retromarginal teeth. Leg measurements: I 6.30 (1.71, 2.24, 1.37, 0.98); II 5.46 (1.55, 1.81, 1.17, 0.93); III 4.93 (1.43, 1.57, 1.16, 0.77); IV 6.54 (1.74, 2.34, 1.53, 0.93). Leg formula: 4123. Opisthosoma oval, pale yellow and hairy, with faint patterns.

***Palp*** (Figs 2A, B, 3C–E). Retrolateral tibial apophysis long and strong. Base of retrolateral tibial apophysis with two small apophyses, extending ventrally and dorsally. Embolus strong, originating at approximately 6-o’clock position, with the anterior part extending inside in the long groove of the conductor. Conductor strong, with J-shaped end.

**One of female paratypes** (SWUC-T-CI-13-02, Fig. 3B) total length 4.66. Prosoma 2.17 long, 1.50 wide; opisthosoma 2.43 long, 1.99 wide. Carapace pyriform, pale yellow, with several rows of setae. Eye sizes and interdistances: AME 0.06, ALE 0.13, PME 0.09, PLE, 0.13; AME–AME 0.01, AME–ALE 0.02, PME–PME 0.09, PME–PLE 0.05, ALE–PLE 0.02. MOA 0.24 long, anterior width 0.13, posterior width 0.30. Clypeus height 0.12. Chelicerae with two promarginal and nine retromarginal teeth. Leg measurements: I 5.29 (1.57, 1.94, 1.07, 0.71); II 4.43 (1.36, 1.49, 0.93, 0.65); III 4.28 (1.23, 1.45, 0.98, 0.62); IV 5.75 (1.68, 1.96, 1.38, 0.73). Leg formula: 4123. Opisthosoma oval, pale yellow and hairy, with distinct patterns.

***Epigyne*** (Figs 2C, D, 3F, G). Atrium large and wide. Copulatory openings located anterior to atrium. Copulatory ducts long and spiral. Spermathecae and secondary spermathecae ball-shaped. Fertilization ducts short.

##### Variation.

Males (*N* = 7) total length 4.10–4.20; females (*N* = 7) total length 3.68–4.66.

##### Distribution.

Known only from the type locality, Chongqing, China (Fig. 8).

#### ﻿ Cicurina yinhe sp. nov.

Taxon classificationAnimaliaAraneaeCicurinidae

39861B57-7EF3-5698-BE37-C9C486CC03B5

https://zoobank.org/6A31F5FE-87D7-447F-A03B-AA57CA90D6E4

**Figure 4.**
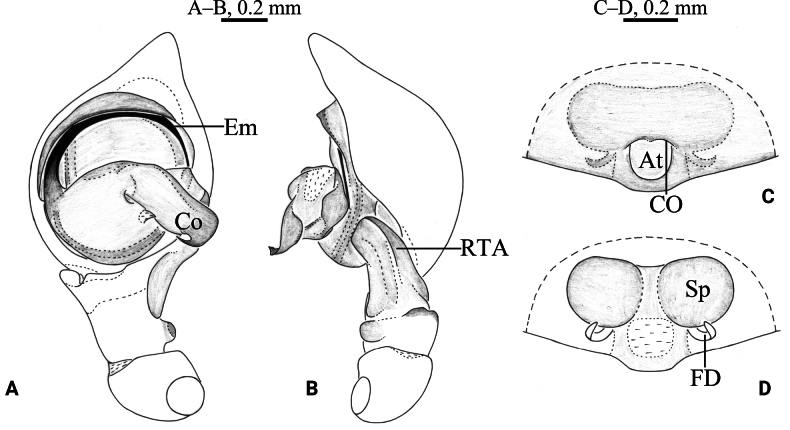
*Cicurinayinhe* sp. nov. holotype male (A, B) and paratype female (C, D). A. Left male palp, ventral view; B. Same, retrolateral view; C. Epigyne, ventral view; D. Vulva, dorsal view. Abbreviation: At = atrium; CO = copulatory opening; Co = conductor; Em = embolus; FD = fertilization duct; RTA = retrolateral tibial apophysis; Sp = spermathecae.

**Figure 5:**
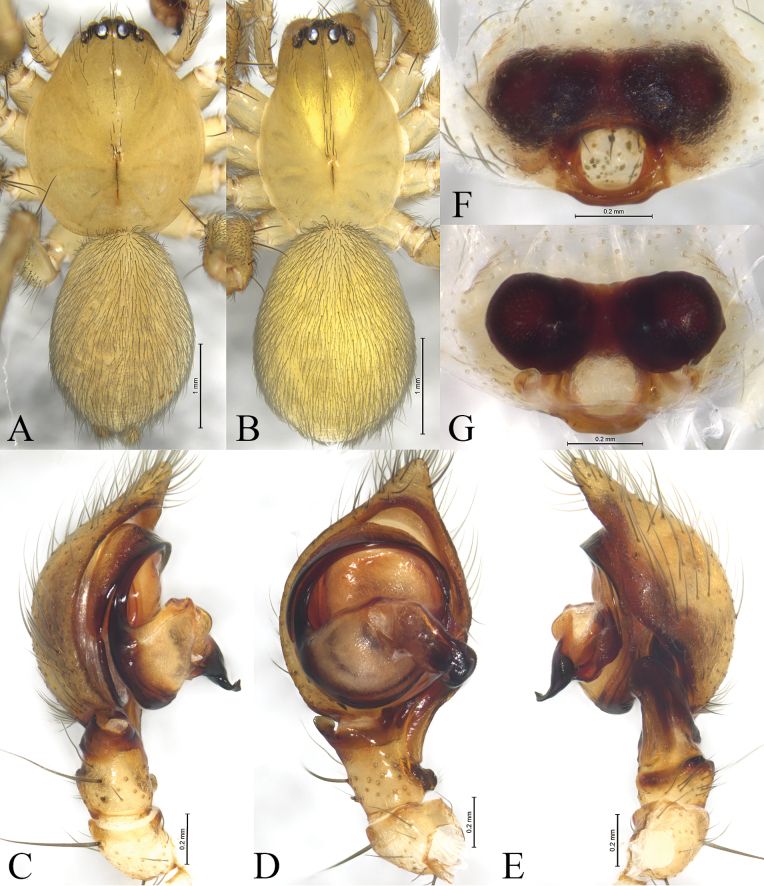
*Cicurinayinhe* sp. nov. holotype male (A, C–E) and paratype female (B, F, G). A. Male habitus, dorsal view; B. Female habitus, dorsal view; C. Left male palp, prolateral view; D. Same, ventral view; E. Same, retrolateral view; F. Epigyne, ventral view; G. Vulva, dorsal view.

Figs 1C , 4A–G , 5A–G , 8 银河洞叶蛛

##### Type material.

***Holotype*** • male (SWUC-T-CI-14-01), China, Chongqing, Beibei, Shuitu Town, Wuji Valley, Yinhe Cave, 29°50'4"N, 106°29'51"E, elev. 550 m, 23 November 2021, L.Y. Wang and T.Y. Ren leg. ***Paratypes***: • 9 males and 3 females (SWUC-T-CI-14-02–13), with same data as for holotype.

##### Etymology.

The specific name refers to the type locality.

##### Diagnosis.

This new species is similar to *C.lichuanensis* Wang, Zhou & Peng, 2019 (Wang et al. 2019: figs 5A–D, 6A–G) in having the similar shaped retrolateral tibial apophysis, the long and strong embolus, the posteriorly located epigynal atrium, and the ball-shaped spermathecae, but differs by the wider and slightly curved retrolateral tibial apophysis (Figs 4B, 5E) (vs. wide and more curved in *C.lichuanensis*), the sharp conductor end (Figs 4A, B, 5C–E) (vs. blunt in *C.lichuanensis*), and the wide epigynal atrium (Figs 4C, D, 5F, G) (vs. wider in *C.lichuanensis*).

##### Description.

**Male holotype** (Fig. 5A) total length 4.92. Prosoma 2.57 long, 2.15 wide; opisthosoma 2.40 long, 1.71 wide. Carapace pyriform and yellow, with a few setae. Eye sizes and interdistances: AME 0.10, ALE 0.14, PME 0.14, PLE, 0.15; AME–AME 0.06, AME–ALE 0.02, PME–PME 0.12, PME–PLE 0.08, ALE–PLE 0.04. MOA 0.35 long, anterior width 0.28, posterior width 0.41. Clypeus height 0.31. Chelicerae with three promarginal and six retromarginal teeth. Leg measurements: I 7.37 (2.02, 2.59, 1.75, 1.01); II 6.81 (1.89, 2.29, 1.63, 1.00); III 6.07 (1.73, 1.92, 1.50, 0.92); IV 7.83 (2.04, 2.49, 2.18, 1.12). Leg formula: 4123. Opisthosoma oval, yellow and hairy, without distinct patterns.

***Palp*** (Figs 4A, B, 5C–E). Retrolateral tibial apophysis wide, with a single fold and truncates apex. The base of retrolateral tibial apophysis with two small apophyses, extending ventrally and dorsally. Embolus strong, originating at approximately 9-o’clock position, with the anterior part extending along the groove of the conductor. Conductor strong, with a small and hooked end.

**One of female paratypes** (SWUC-T-CI-14-02, Fig. 5B) total length 4.40. Prosoma 2.25 long, 1.55 wide; opisthosoma 2.27 long, 1.69 wide. Carapace pyriform and yellow, with a few setae. Eye sizes and interdistances: AME 0.11, ALE 0.13, PME 0.12, PLE, 0.14; AME–AME 0.06, AME–ALE 0.02, PME–PME 0.11, PME–PLE 0.07, ALE–PLE 0.02. MOA 0.37 long, anterior width 0.27, posterior width 0.36. Clypeus height 0.13. Chelicerae with three promarginal and six retromarginal teeth. Leg measurements: I 5.29 (1.75, 2.19, 1.28, 0.77); II 4.43 (1.51, 1.84, 1.16, 0.76); III 4.28 (1.43, 1.54 1.15, 0.69); IV 5.75 (1.84, 2.12, 1.59, 0.85). Leg formula: 4123. Opisthosoma oval, yellow and hairy, without distinct patterns.

***Epigyne*** (Figs 4C, D, 5F, G). Atrium oval. Copulatory openings located on the shoulders of atrium. Spermathecae large and ball-shaped. Fertilization ducts hook-shaped.

##### Variation.

Males (*N* = 8) total length 4.13–4.92; females (*N* = 3) total length 3.94–4.40.

#### ﻿ Cicurina zhangfui sp. nov.

Taxon classificationAnimaliaAraneaeCicurinidae

9D669241-D5E8-5B27-823A-4914B5CF22D5

https://zoobank.org/CDD0F574-BFE7-45D5-8FE5-9EF4D643543E

**Figure 6.**
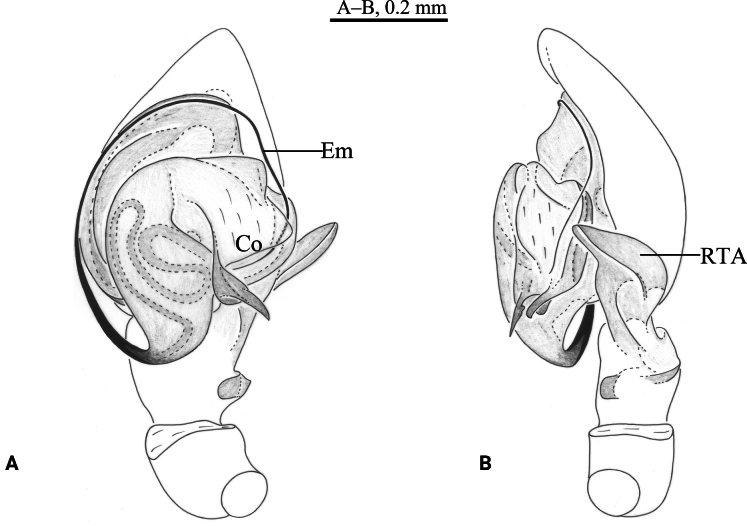
*Cicurinazhangfui* sp. nov. holotype male (A, B). A. Left male palp, ventral view; B. Same, retrolateral view. Abbreviation: Co = conductor; Em = embolus; RTA = retrolateral tibial apophysis.

**Figure 7:**
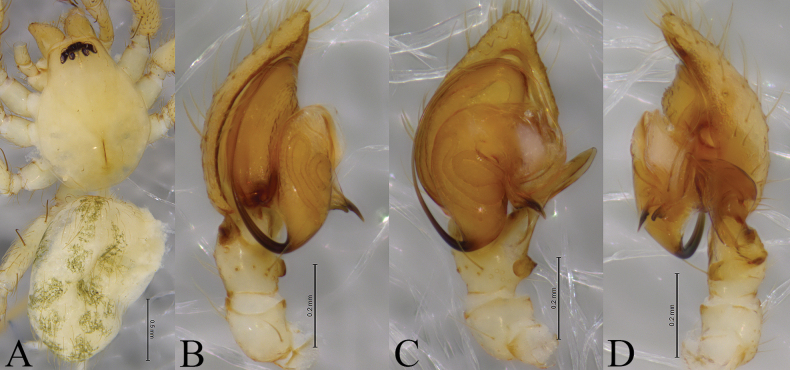
*Cicurinazhangfui* sp. nov. holotype male (A–D). A. Male habitus, dorsal view; B. Left male palp, prolateral view; C. Same, ventral view; D. Same, retrolateral view.

**Figure 8:**
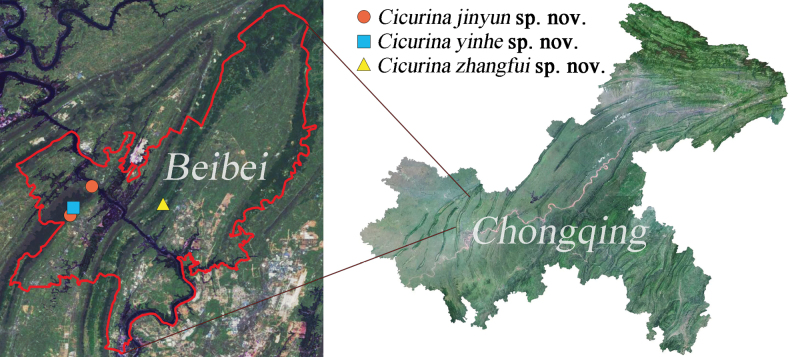
Distribution records of three *Cicurina* species in Chongqing, China.

Figs 6A, B , 7A–D , 8 樟福洞叶蛛

##### Type material.

***Holotype*** • male (SWUC-T-CI-15-01), China, Chongqing, Beibei, Jinyun Mountain National Nature Reserve, 29°49'58"N, 106°23'2"E, elev. 804 m, 7 January 2011, J. Yang and S.Y. Xu leg. ***Paratypes***: • 1 male (SWUC-T-CI-15-02), with same data as for holotype; • 1 male (SWUC-T-CI-15-03), Jinyun Mountain National Nature Reserve, 29°49'58"N, 106°23'2"E, elev. 804 m, 15 February 2011, L. Li and M.Y. Liu leg.

##### Etymology.

The specific name of this new species is dedicated to the Chinese arachnologist Prof. Zhangfu Chen in honor of the first description of a *Cicurina* species in China.

##### Diagnosis.

The male of this new species is similar to that of *C.avicularia* Li & Wang, 2017 (Li and Wang 2017: figs 62A, B, 63A, B, 65A–D, 66E) in having the similar shaped retrolateral tibial apophysis, and the slender and filiform embolus, but differs by the weakly bifurcated conductor end (Figs 6A, B, 7B–D) (vs. with strongly bifurcated in *C.avicularia*).

##### Description.

**Male holotype** (Fig. 7A) total length 2.65. Prosoma 1.24 long, 0.92 wide; opisthosoma 1.37 long, 0.95 wide. Carapace pyriform and pale yellow, with sparse setae. Eye sizes and interdistances: AME 0.05, ALE 0.06, PME 0.06, PLE, 0.06; AME–AME 0.03, AME–ALE 0.02, PME–PME 0.07, PME–PLE 0.05, ALE–PLE 0.02. MOA 0.15 long, anterior width 0.12, posterior width 0.17. Clypeus height 0.09. Chelicerae with three promarginal and six retromarginal teeth. Leg measurements: I 3.26 (0.94, 1.11, 0.67, 0.54); II 2.93 (0.86, 0.98, 0.60, 0.49); III 2.69 (0.80, 0.79, 0.62, 0.48); IV 3.33 (0.95, 1.08, 0.81, 0.49). Leg formula: 4123. Opisthosoma oval, pale yellow and sparsely hairy, with distinct patterns.

***Palp*** (Figs 6A, B, 7B–D). Retrolateral tibial apophysis wide, with a single fold and rounded end. Embolus slender and filiform, originating at approximately 6-o’clock position, with the anterior part extending along the long groove of the conductor. Conductor short and strong, with a weakly bifurcated end.

**Female.** Unknown.

##### Variation.

Males (*N* = 3) total length 2.65–2.77.

##### Distribution.

Known only from the type locality, Chongqing, China (Fig. 8).

## Supplementary Material

XML Treatment for Cicurina jinyun

XML Treatment for Cicurina yinhe

XML Treatment for Cicurina zhangfui

## ﻿Additional information

### Use of AI

No use of AI was reported.

### Author ORCIDs

Hui-Yi Chen https://orcid.org/0009-0006-0449-7300

Lu-Yu Wang https://orcid.org/0000-0002-5250-3473

Zhi-Sheng Zhang https://orcid.org/0000-0002-9304-1789

Feng Zhang https://orcid.org/0000-0002-3347-1031

### Data availability

All of the data that support the findings of this study are available in the main text.
